# Monitoring Re-Growth of Invasive Plants Using an Autonomous Surface Vessel

**DOI:** 10.3389/frobt.2020.583416

**Published:** 2021-01-22

**Authors:** Robert Codd-Downey, Michael Jenkin, Bir Bikram Dey, James Zacher, Eva Blainey, Peter Andrews

**Affiliations:** ^1^ Department of Electrical Engineering and Computer Science, York University, Toronto, ON, Canada; ^2^ Judy Dan Research and Treatment Centre, Toronto, ON, Canada; ^3^ Farlain Lake Community Association, Tiny, ON, Canada

**Keywords:** computer vision, object detection, dataset, Underwater, robotics, autonomous surface vessel, plant monitoring

## Abstract

Invasive aquatic plant species, and in particular Eurasian Water-Milfoil (EWM), pose a major threat to domestic flora and fauna and can in turn negatively impact local economies. Numerous strategies have been developed to harvest and remove these plant species from the environment. However it is still an open question as to which method is best suited to removing a particular invasive species and the impact of different lake conditions on the choice. One problem common to all harvesting methods is the need to assess the location and degree of infestation on an ongoing manner. This is a difficult and error prone problem given that the plants grow underwater and significant infestation at depth may not be visible at the surface. Here we detail efforts to monitor EWM infestation and evaluate harvesting methods using an autonomous surface vessel (ASV). This novel ASV is based around a mono-hull design with two outriggers. Powered by a differential pair of underwater thrusters, the ASV is outfitted with RTK GPS for position estimation and a set of submerged environmental sensors that are used to capture imagery and depth information including the presence of material suspended in the water column. The ASV is capable of both autonomous and tele-operation.

## 1 Introduction

Invasive aquatic plant species are non-native plants that are spread by international trade as well as local human and animal transport. These plants invade lakes and oceans preventing domestic plants from growing through either light or nutrient starvation. Many invasive species have no value to local wildlife which puts addition pressure on the remaining plant life. This pressure can migrate up the food chain. Dense growth of invasive species can be detrimental to recreational water-sports, commercial diver operations and ruin the natural beauty of the affected regions.

Eurasian Water-Milfoil *myriophyllum spicatum* is an invasive species now found in the St. Lawerence river, the Great Lakes and many inland lakes in Canada (EWM Ministry of Natural Resources and Forestry. (2020)). Native to Europe, Asia and Africa it was introduced to North America in the 19th centrury and is now found widely across North America. It has many deleterious effects on the environment it invades; as an aggressive competitor to native plants it rapidly decreases bio-diversity. At the end of its annual life-cycle in the fall it begins to decompose and reduces oxygen levels in the water. During the summer months its growth inhibits recreational water-sports, impedes boat traffic in commercial harbors and creates regions of stagnant water where mosquitoes can proliferate. Within Ontario and Canada more generally there is a concerted effort to monitor and when practical reduce the spread of EWM.

A key problem with EWM is that is spreads through vegetative reproduction. That is, it is spread through plant fragmentation and root expansion. Thus even a very small piece of this plant can re-root and form a new colony. It is a fragile plant and easily fragmented. Furthermore, naive attempts to remove the plant, (e.g. hand cutting or driving a power boat through a surface colony) will only result in plant fragments, each of which can form a new colony. If not controlled it can form thick floating mats of vegetation.

**FIGURE 1:**
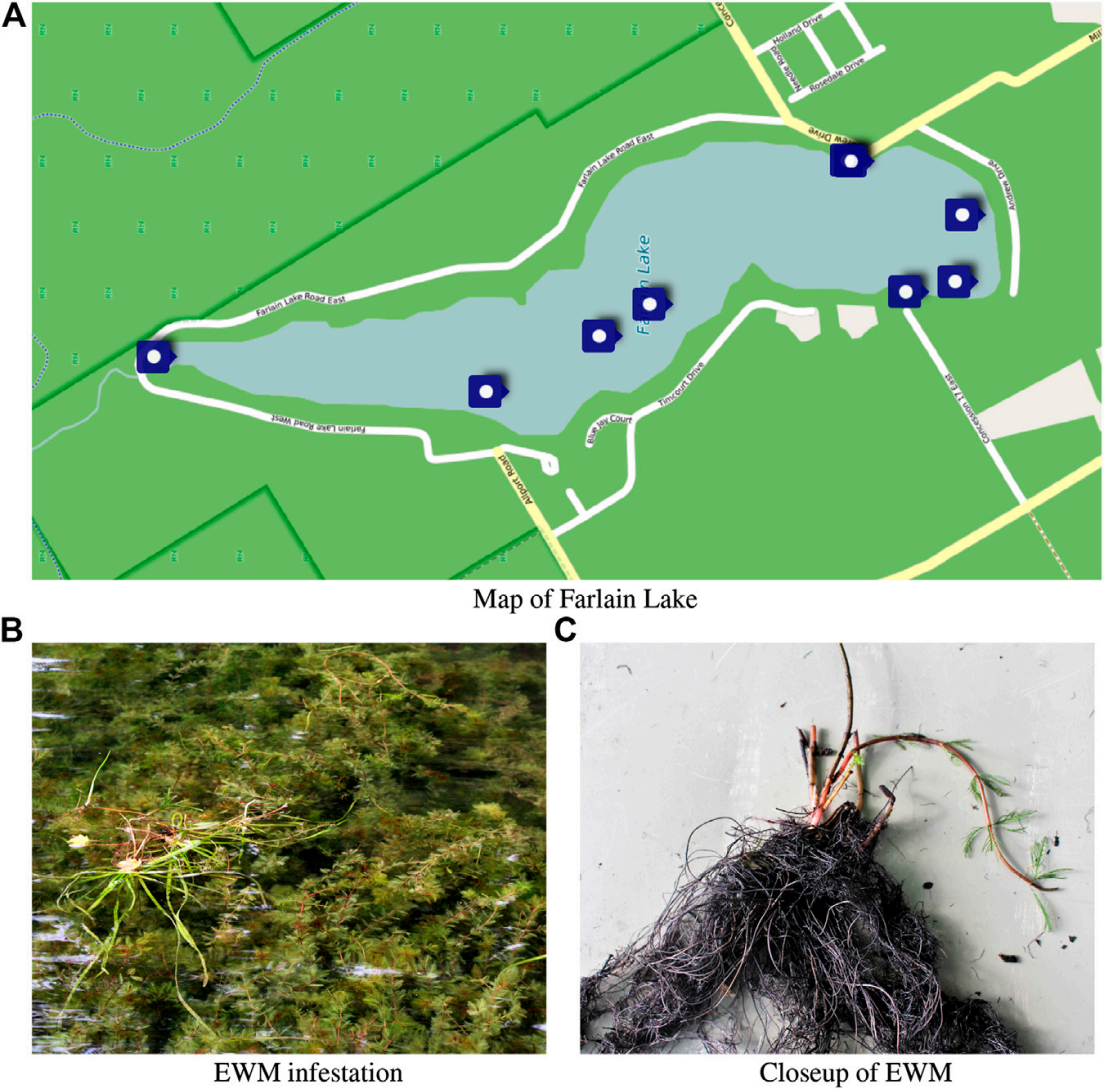
Known concentrations of EMW on Farlain Lake as of summer 2020 and closeup views of the plant **(B)** and **(C)** appear with the kind permission of the Farlain Lake Community Association.

Farlain Lake in Ontario, Canada has become home to an EWM infestation and the local community is currently fighting to prevent it from taking over the lake (Farlain Lake Community Association. (2020)). As Farlain Lake is not river fed the most likely cause for the introduction of EWM into the lake is from personal watercraft that have been transported from an infected lake into Farlain Lake. The local community is engaged in monitoring the growth of the plant and its presence in the lake. Figure 1 shows known concentrations of EWM in Farlain Lake as of summer 2020 along with a sample of the plant.

Commonly found in shallow waters (1–3 m deep), the root system of EWM can be found in much deeper water due to its ability to grow up to 5–6 m tall (CABI. (2019)). Dealing with this plant from the surface with human-operated vessels is difficult as precise localization and the 3–6m depth to the plant’s base are significant complications. This makes this task suitable for autonomous surface vessel access given its close proximity to the surface and limited growth area near the shore.

Monitoring the infestation of EWM within a body of water is a complex task. The plant grows over a range of different depths and the turbidity of lakes in Ontario can make it difficult to monitor infestation from the surface. It can be quite easy to monitor infestations near the shore, as here the water column in shallower and individuals (home and cottage owners) can monitor the infestation by simply wandering into the water. Monitoring infestation at depth is more difficult and expensive. SCUBA divers can be deployed to seek out infestations but localizing this information into an earth-based coordinate frame is complicated by the lack of good underwater localization under such conditions.

Here we describe various methods being tested to monitor and treat the threat that Eurasian Water-Milfoil poses to Farlain Lake and the surrounding community and efforts to use an autonomous surface vessel to help automate the task. A key question in treating EWM is understanding the efficacy of different approaches and how combinations of different treatments might be combined to deal with an infestation. Given the need to obtain quantitative geo-tagged measurements of the level of the infestation, we are exploring how these measurements might be augmented through the use of an autonomous surface vessel.

## 2 Invasive Aquatic Plant Monitoring and Removal

The traditional and time intensive approach to monitoring plant infestation is through surface monitoring using commodity GPS sensors and a group of dedicated volunteers. This was the approach used to obtain the map of infestations shown in Figure 1. This approach “works” but it suffers from the need to utilize a number of volunteers to monitor the lake, the lack of a standard measure of the level of the infestation, uneven sampling of the lake, and relatively poor quality localization information. Never the less this approach can be used to identify gross infestation sites which can then be sampled, (e.g. by SCUBA divers) to determine the level of the infestation.

Once an infestation has been identified there exist a number of methods that have been developed to remove invasive aquatic plants from their environment (see Gettys et al. (2014)). Each of these methods has its own benefits and caveats. Here we describe the methods being used and evaluated for their ability to effectively remove Eurasian Water-Milfoil from Farlain Lake.

### 2.1 Hand Harvesting

Hand harvesting involves physically uprooting each plant and collecting them in bags to be properly destroyed. The process of harvesting the plant is quite complex as harvesting the plant without also collecting the root system is not effective. Nor is harvesting the root and not properly collecting the entire plant. Proper handling and disposal is necessary to prevent re-growth. Even small pieces of the plant can proliferate and so harvesting must be careful to not break the plant into separate parts that in time can become full plants. That being said, hand harvesting can be an effective solution when dealing with small patches in areas close to shore. When dealing with larger patches in deeper water SCUBA divers are needed to harvest. This can quickly become arduous and cost prohibitive as hand harvesting is a very slow process. Whether harvesting in the shallows or at depth it is necessary to provide a mechanism to capture broken off portions of the plant and to properly capture and dispose of all plant material that is harvested. That being said, hand harvesting has proven effective in certain domains. For example Kelting and Laxson. (2010) describes a successful multi-year effort to control EWM in Adirondack Park.

### 2.2 Benthic Mats

**FIGURE 2:**
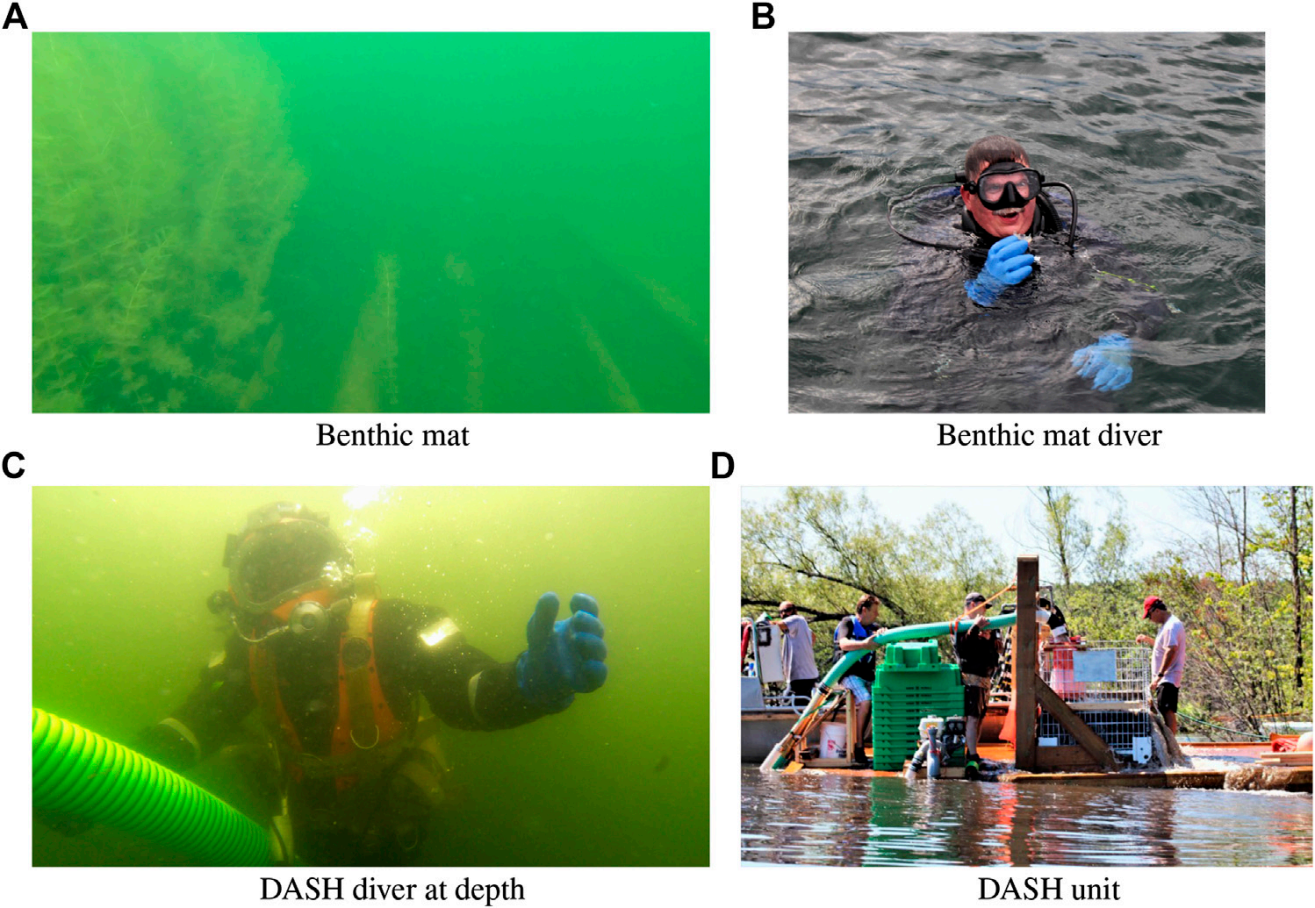
Treating Eurasian Water-Milfoil. Two different mechanisms are shown here **(A)** and **(B)** illustrate the application of Benthic mats **(A)** shows a Benthic mat applied to the bottom. On the right side of the image the EWM has been covered with the mat. On the left side is shown the growth beyond the mat **(B)** shows a diver dealing with the mat. A key problem in deploying the mats is communicating with operators on the surface delivering the mats to divers operating at depth **(C)** shows a diver operating the DASH system at depth. This venturi pump is supported by a large operating unit on the surface shown in **(D)**.

Benthic mats are large sheets or mats that are laid over the bottom of a bed of water to prevent and inhibit growth (see Figure 2A, B. These mats block sunlight thus subverting the process of photosynthesis. Mats can be manufactured of various materials with permanent, (e.g. plastic Mayer. (1978)) and biodegradable, (e.g. jute or burlap Caffrey et al. (2010)) being common. The primary advantage of the use of biodegrabable material is that they degrade over time and if properly applied do not leave a residue in the lake. In the application in Farlain Lake biodegradable mats are placed over infected areas and left there. Mats are secured to the lake bed using weight bags made of the same biodegradable material.

One concern with benthic mats is that they are not selective and they affect all flora and fauna that reside under the mat. The Farlain project uses burlap mats that decay in 2–3 years and permit some native plants (and potentially invasive plants) to grow on the sediment on top of the mat. A recent visit to a benthic mat site on Farlain Lake showed over 30 cm of sediment over the mats placed only a few years ago. Figure 2A shows the efficacy of the approach. On the right hand side of the image the bottom on the lake is covered with benthic mats. On the left hand side EWM can be seen growing to over 1m over the height of the benthic mat material.

### 2.3 Diver Assisted Suction Harvester (DASH)

The diver assisted suction harvester (DASH) system (Tucker. (2017)) is essentially a boat mounted vacuum designed to filter out and remove vegetation. The suction end of the hose is operated by a SCUBA diver to prevent the system from capturing native plant species as much as possible. Figure 2C,D shows the system in operation on Farlain lake. While the DASH system is fast it carries the large overhead cost of installing the system on a suitable boat. Other concerns include that hard sediment at the lake bed can cling to root systems preventing full extraction, soft sediment can be lifted into the intake hose relocating and possibly killing bottom dwelling organisms.

The primary concerns with DASH systems relate to cost and ensuring that the process does not damage native plants and the cost of applying the technology. As the diver remains at depth this reduces the risk to the diver, but at the same time the suction house may capture much more than the intended plant material. One major advantage of DASH systems is that they can efficiently extract and bag two cube meters of material every 5 min. This is a rate that is approximately 50 times faster than hand harvesting.

### 2.4 Chemical Herbicides

There are many chemicals that can be used as effective herbicides to kill or prevent further growth of invasive aquatic plant species. Some of these chemicals pose risks to humans and other wildlife and in Canada their use is regulated by Fisheries and Oceans Canada. Further regulations can be imposed by provinces and territories (Fisheries and Canada. (2019)). Due to safety concerns and regulations in Ontario, the Farlain lake association has only been granted limited access to apply herbicide during specific times and locations around the spawning cycle of the local fish. However multiple treatments are necessary for a chemical herbicide approach to be effective.

### 2.5 Integrated Approaches

Although each of the above approaches can be applied separately it is also possible to apply different mechanisms in combination to deal with EWM infestation. A key problem then becomes one of monitoring the efficacy of different approaches (and a combination of approaches) to ensure that the techniques being applied are effective and are the best for the specific environment. However, monitoring the current state of the environment is difficult. The use of volunteers with variable levels of attention to detail and different sensors with varying levels of accuracy limits the overall estimation of the efficacy of different approaches and combination of approaches. A key observation is this: in order to deal with an infestation such as EWM it is critical to be able to monitor the state of the infestation in a systematic and ongoing manner. A monitoring procedure that will be difficult with volunteers but a process that would be ideally suited for for a robotic application.

## 3 Monitoring the Infestation

In order to properly evaluate the effectiveness of these invasive plant removal methods three separate infected regions have been chosen on the lake. Within each region a different method is being used and evaluated. Evaluation can be accomplished through various means, but perhaps the most effective method at present involves the use of SCUBA divers. Visual surveys of each area are conducted by SCUBA divers and recorded on a video camera for later observation. Unfortunately camera jitter and the movement of vegetation in the water can affect the perception of true motion along the survey path, thus accurate estimation of growth can be difficult. One approach here is to mount markers on the lake bed so that measurements from year to year can be related to these fiducial markers. However such markers must be maintained and this incurs ongoing operational cost. Monitoring the state of the infestation from the surface requires vehicles equipped with accurate GPS hardware to transit the body of water on a regular basis and to capture information from depth about the state of the lake. This task seems ideally suited for an appropriately equipped autonomous vehicle. In order to provide such coverage we have developed Eddy, an autonomous surface vessel (ASV).

There is a long history of the development of autonomous, or at least remotely controlled, surface vessels. Perhaps the earliest is Tesla’s wireless radio controlled boat from the 1890’s Marincic and Budimir. (2008). There are examples of remote-controlled boats being used as a weapon. In the First World War, for example, Germany developed the *Fernlenkboot* or FL-boat (also known as a distance-controlled explosive boat). Following the Second World War a number of navies developed autonomous surface vessels, for a range of tasks from remote controlled targets a well as for tasks such as minesweeping. A history of Unmanned surface vessels (USV) and ASV designs can be found in Liu et al. (2016) and Bertram. (2008).

ASV’s have been built to perform a range of bathymetric tasks, (e.g. Brown et al., 2010; Mousazadeh et al., 2017), reef monitoring, (e.g. Romano and Duranti, 2012; Pizarro et al., 2017), disaster monitoring and assistance (see Jorge et al., 2019 for a review), pollution monitoring, (e.g. Vasilj et al., 2017) and commercial infrastructure monitoring, (e.g. Han et al., 2015) to note but a few such applications. ASV’s have been developed that can perform this task individually or as part of a fleet, (e.g. Mojiri Forooshani and Jenkin, 2015) and even as part of a multi-platform team, (e.g. Shkurti et al., 2012; Pieterkosky et al., 2017). A number of navies and coast guards/police forces have also begun to develop and deploy ASV’s and commercial platforms are available from a number of companies including ASVGlobal and Clearpath. Devices have been developed to run under a range of different operational models from teleoperational control to fully autonomous.

For the EWM monitoring task a small scale fully autonomous device was required that could be deployed without specialized equipment and that could operate autonomously for short duration (1 h or less) missions to monitor previously identified portions of the lake. As no commercial device existed at the time we developed a custom platform (Eddy) as described below.

### 3.1 Eddy: The Autonomous Surface Vessel

**FIGURE 3:**
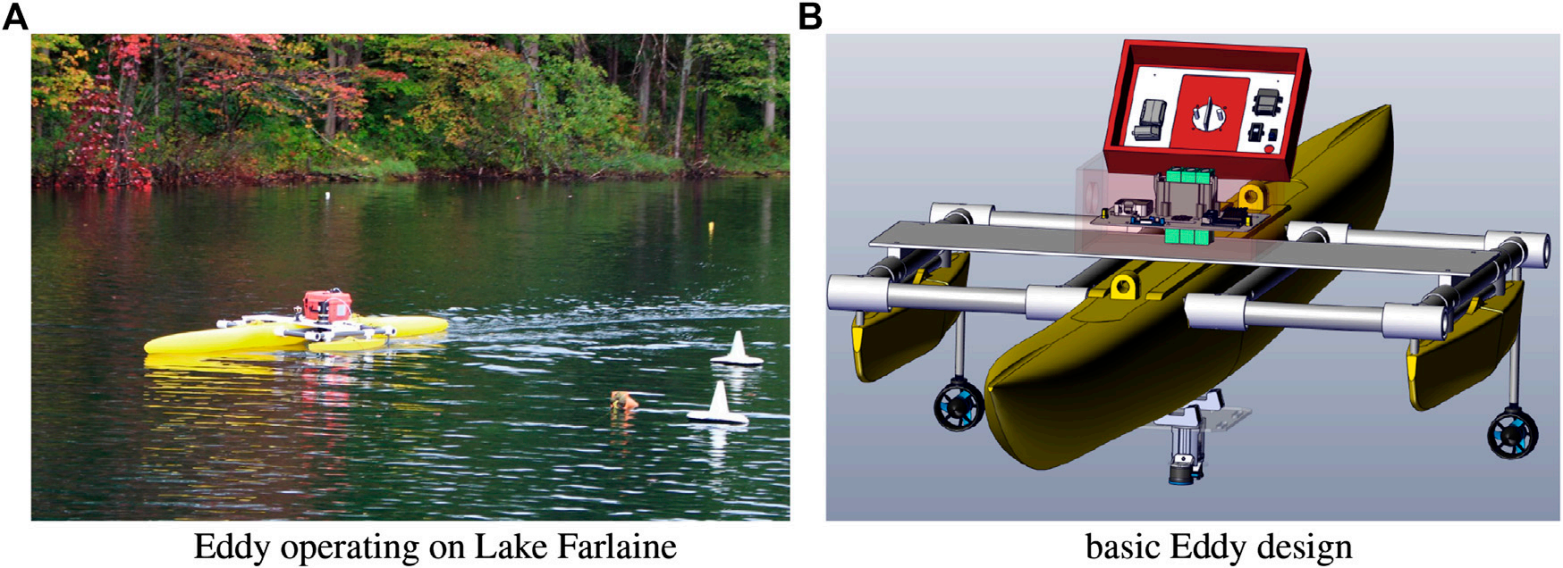
Eddy **(A)** shows Eddy deployed on Lake Farlain **(B)** shows a rendering of the 3D CAD model of all the physical and electronic components of the Eddy robotic platform. Eddy is a monohull design with two outriggers. Two thrusters in a “differential drive” configuration provide position and orientation control. A detachable hull-mounted sensor platform provides depth and visual information about the infestation below the vessel while RTK GPS coupled with an IMU provide vehicle state information. Eddy is designed to be easily disassembled into small hand-portable components that can be easily deployed to different sites. Eddy is designed to be disassembled into small hand portable components that can be easily deployed to different sites.

Eddy (see Figure 3) is a monohull design with two outriggers to provide stability. It utilizes a differential drive locomotion model provided by two BlueRobotic T200 thrusters. From the outset it was recognized that Eddy would have to be easily hand portable and in order to allow operations to take place at some distance from home, to be easily portable by commercial air. This last constraint requires the robot to be disassembled into component parts that are light and can survive the rigors of international air travel. It is also possible that the vessel could be damaged during transport and thus large components that might not fit in hand luggage should be easily replaceable away from the resources of the lab. With the exception of the hull and outriggers, the rest of Eddy’s structure is built from easily available PVC pipes that are joined with easily removable pins. Eddy can be assembled and disassembled into its components in a few minutes by one person.

**FIGURE 4:**
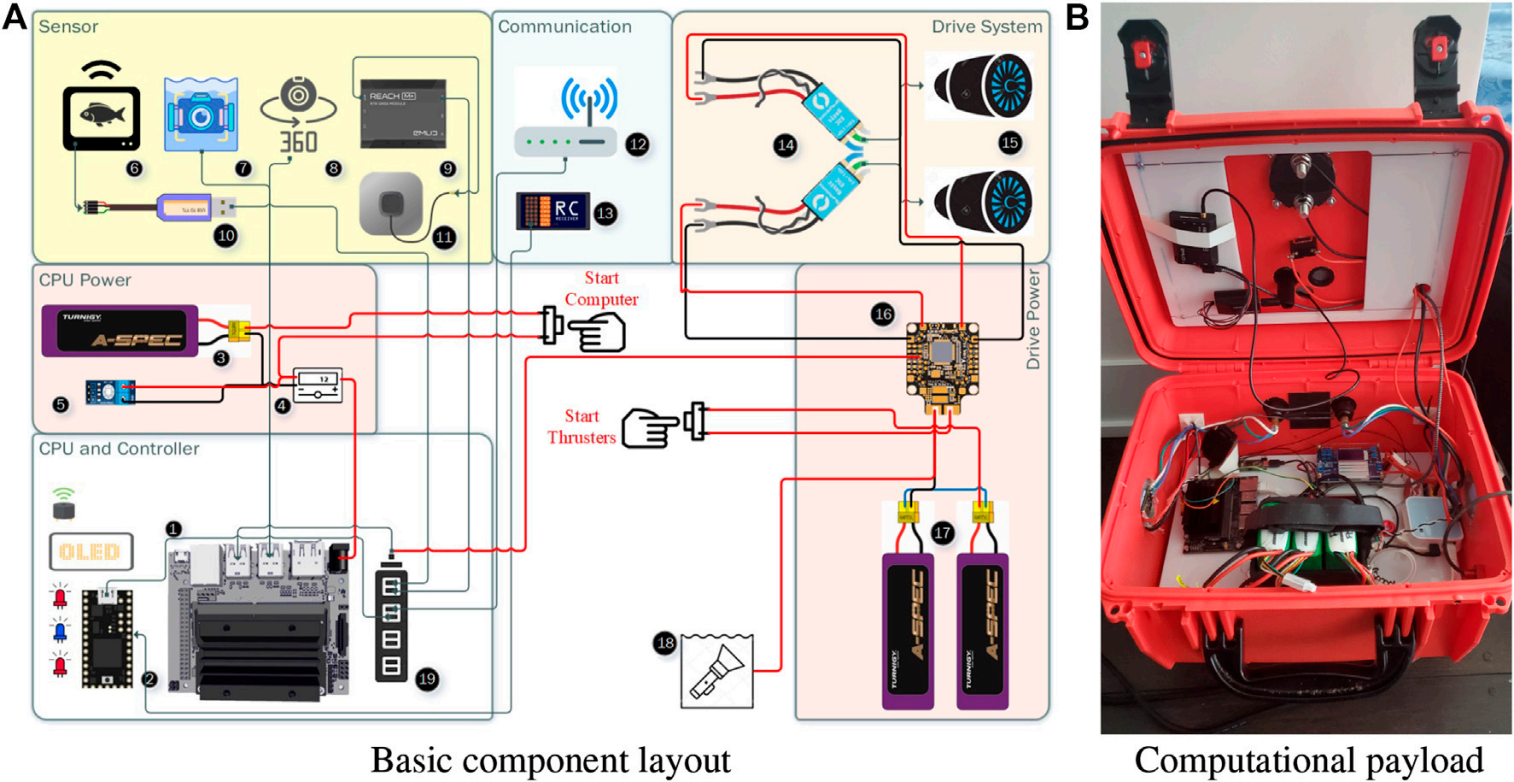
Eddy’s compute and power infrastructure is confined to a single water-tight control box mounted on the frame. The box is connected via two quick-disconnect pins and is operated closed **(A)** a schematic of Eddy’s computational, sensing and power infrastructure **(B)** the components that exist within Eddy’s control box.

**FIGURE 5:**
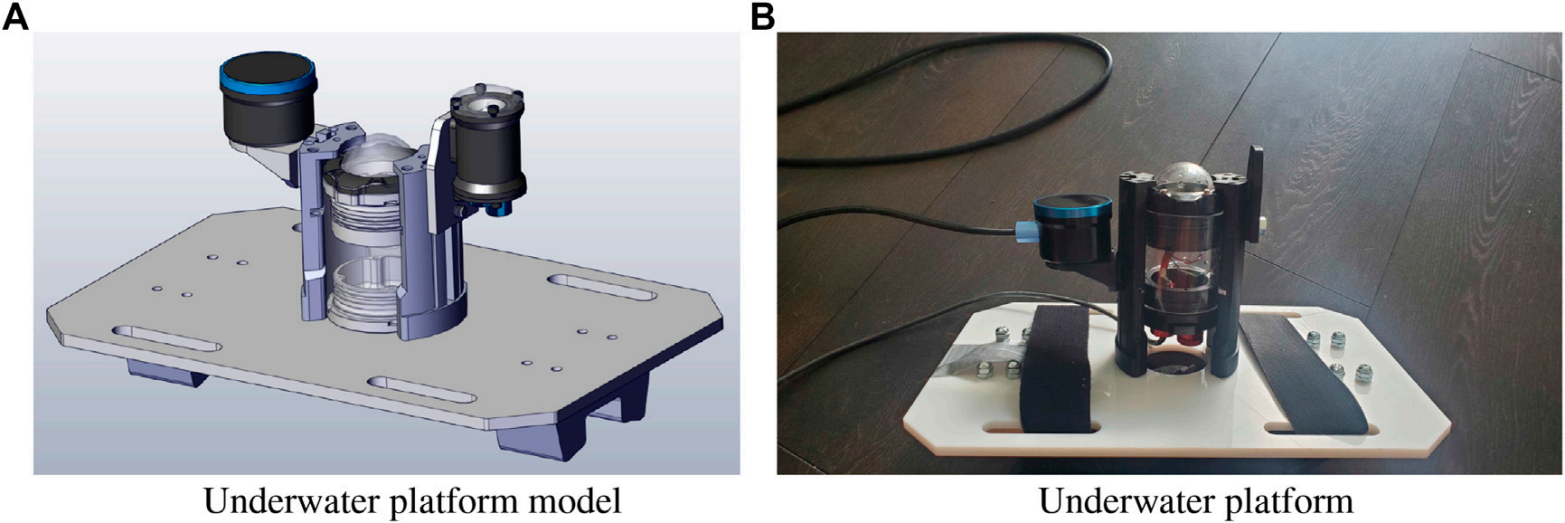
Underwater sensor infrastructure. Sensors within Eddy are located within an IP67 box mounted on the top of the vehicle or are mounted below the vehicle on a sensor platform affixed to the underside of the monohull **(A)** A model of the sensor platform mounted below the hull (here shown inverted) **(B)** The actual sensor platform with the computer controllable light removed. The sensor platform is attached to the hull using straps.

Given the need to be able to easily disassemble Eddy for transport all components including the thrusters, compute/power infrastructure and sensors are also designed to be easily separated from the robot. Figure 4 provides details of this infrastructure on Eddy. All components that do not require to be submerged are located in or on the water-tight control box that is mounted on top of the hull (the “red box” seen in Figure 3). Figure 4A provides a schematic of the power/sensor/control infrastructure of Eddy and Figure 4B shows the infrastructure arrayed within the control box. Eddy’s thrusters and underwater sensors are connected to the ontrol box using waterproof cables. Given Eddy’s deployment for monitoring EWM below the surface Eddy is equipped with sensors that monitor the water column. These are mounted on a plate that mounts via a strap to the hull. Figure 5 shows this platform. Figure 5A shows a drawing of the fully populated underwater sensor platform with camera, ping echo sounder and controllable light source. Figure 5B shows the realized sensor platform with the controllable light source removed.

Eddy’s electrical and control components are sketched in Figure 4. The robot utilizes separate power systems for the thrusters and compute/sensor infrastructure to minimize power disruption that might be caused by the thrusters. Onboard computing is provided by an NVIDIA Jetson which is supplemented by a Teensy Microcontroller for time critical functions. One critical function performed by the Teensy is to implement a deadman switch based on an RC Controller signal. Should the RC signal fail, the robot stops and a separate RC channel allows a remote supervisor to take over vehicle control if necessary.

In terms of sensors, Eddy is equipped with an Emlid Reach M + RTK GNSS/IMU module and an onboard IMU. In order to monitor its environment Eddy is equipped with a BlueRobotics single beam echo-sounder and a BlueRobotics downward facing low-light HD camera paired with a BlueRobotics SubSea 1,500 lumen LED. This camera/light pair is used to image any EWM below the vehicle. The USV runs ROS (Quigley et al. (2009)) and can be operated autonomously. A system simulator has been constructed in Gazebo (Koenig and Howard. (2004)) to enable off-robot testing and visualization. For the data presented here the robot was teleoperated.

### 3.2 Navigation

Eddy uses the ROS navigation stack to provide GPS waypoint navigation. Integration with the navigation stack requires a number of basic capabilities that must be implemented on the platform including: Odometry model, Velocity Controller, Obstacle Detection Sensors and Environment Map. To bridge the gap between the Cartesian coordinate space that the navigation stack operates in to the GPS coordinate space we use the ROS gps_goal package which initializes the origin of the Cartesian space to the first GPS position received.

#### 3.2.1 Odometry Model

The dynamics of an aquatic vehicle are much more difficult to model than their terrestrial counterparts due to a number of external factors that are difficult to estimate and model. These include buoyancy and wind/wave action. Given frequent assembly/disassembly of the vehicle even an accurate estimation of vehicle inertia would difficult. Given these and other complications a simple differential drive model of the vehicle would be far too inaccurate to provide a reasonable measure of the vehicle’s motion. To overcome this we do not model the motion of the vehicle directly, instead we rely on the onboard RTK GPS and IMU to measure the vehicle’s state directly. The data from these sensors are fused together using an EKF to provide a two dimensional odometry model using the robot_localization package in ROS. A very simple model of the effective force of each thruster is used within a vehicle plant model.

#### 3.2.2 Velocity Controller

Unlike motor controllers on terrestrial vehicles the RPM of the BlueRobotics T200 thrusters directly corresponds the force exhibited on the vehicle instead of the velocity of the thruster joint. Low-level control of the robot is provided via a “/cmd_force” topic which takes the form of a geometry_msgs/Wrench message which has separate components for linear and angular forces. The PWM values to produce the required force from the left and right thruster is calculated by using the closest values in the lookup table provided by BlueRobotics and interpolating between these values. Velocity control is provided by two PID controllers for which linear and angular velocity measurements are obtained from the sensor fused odometry model.

#### 3.2.3 Obstacle Detection Sensors

Normally the ROS navigation stack utilizes sensor_msgs/LaserScan messages produced from a 2D LIDAR sensor or sensor_msgs/PointCloud messages produced from 3D LIDAR sensor or stereo/RGBD cameras to perform local obstacle detection. These measurements inform the local planner to provide obstacle avoidance. Fortunately Eddy can operate under the assumption that there are no obstacles in the environment so these sensor are unnecessary to the current problem.

#### 3.2.4 Map Server

The navigation stack utilizes an occupancy grid map to inform the associated global and local planners, without such a map the entire environment is assumed to traversable. Under this assumption any GPS waypoint needs to be selected carefully so that the robot does not collide with the shore or other structures.

### 3.3 Sensing

**FIGURE 6:**
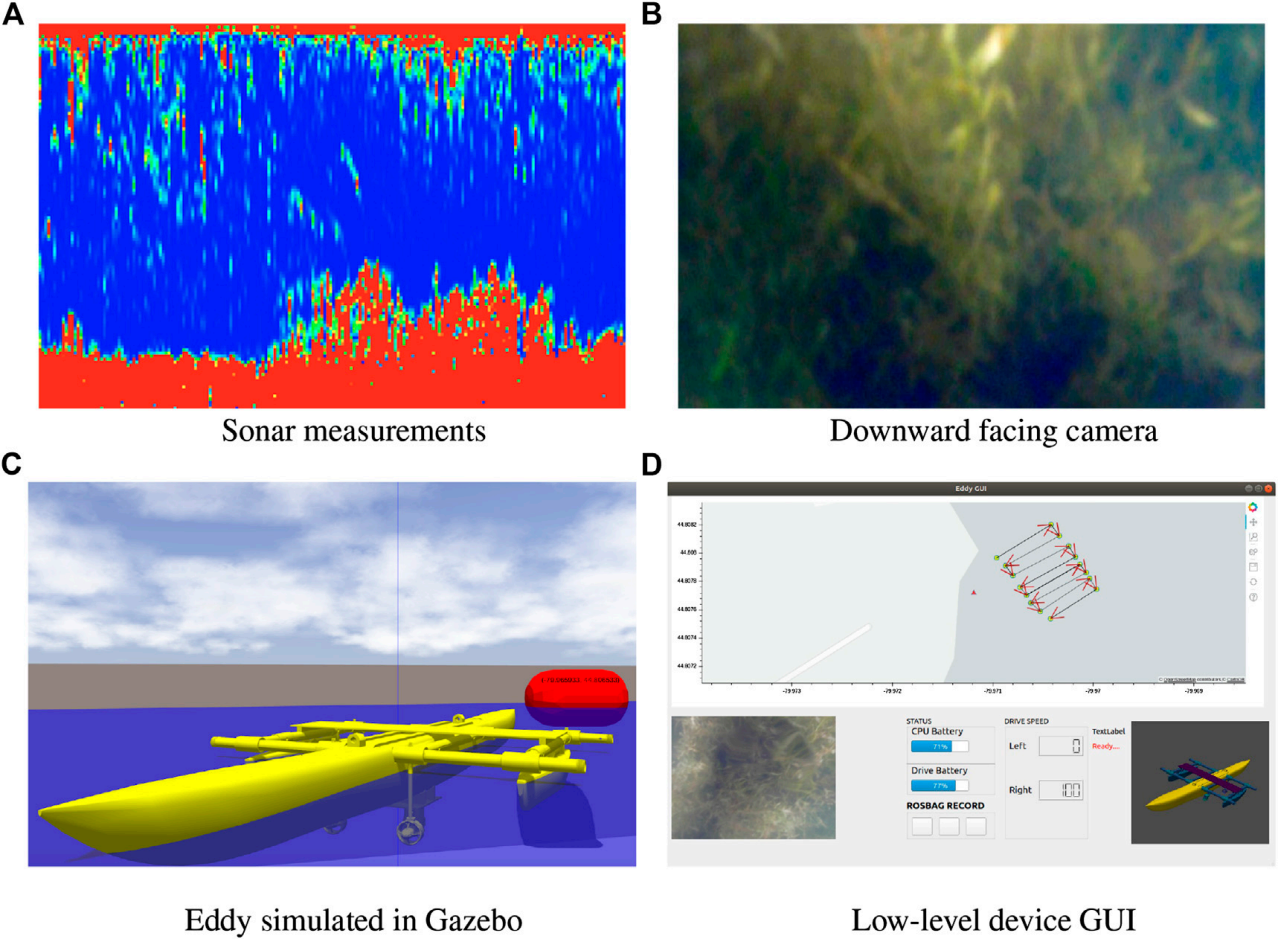
Eddy sensor measurements **(A)** a windowed view of the data from the on board sonar sensor displaying response from EWM in the water column **(B)** an image captured from the on board low-light HD camera showing an EWM infestation **(C)** an image of the robot from a Gazebo simulation used for testing **(D)** GUI for driving Eddy; top window shows the location of the boat in real-time and the waypoints, bottom left shows the real time camera feed and center shows battery status, rosbag status and bottom right shows boat roll-pitch-yaw of the vehicle.

Eddy has two on board sensors that are used to measure and evaluate the level of plant infestation. The SONAR sensor is used to calculate the depth of the water column and provide insight into the density of vegetative matter at various depths below the ping sensor. The BlueRobotics Ping SONAR API allows the sensor to be configured automatically which changes the scan range and gain based on environmental conditions. This setting helps is useful in aquatic environments where the bathymetry of the lake is unknown prior to deployment. This can result in historical data from having different scales from current measurements. Fortunately this setting does maintain a accurate depth estimate when large changes in depth occur in the water column. The downward facing camera is especially useful for imaging the extent of the infestation as well as differentiating between invasive and non-invasive plant species. The accompany LED is used to balance illumination in the camera frame. When the overall illumination in the camera image falls below a programmed threshold the LED is powered to a level to reestablish the desired illumination. Figure 6A shows a sample return signal from the sonar sensor (vertical) as a function of time (horizontal). The surface of the water is at the top of the image. Blue portions of the signal represent open water. This signal allows estimation of the current water depth as well as material found in the water column. Figure 6B shows a sample image of the downward facing camera imaging EWM. Figure 6C shows the Gazebo simulator of the robot. Currently these sensor measurements are only analyzed by researchers after being recorded by the robot alongside IMU and GPS measurements and compiled into a geo-tagged format that can be viewed by software such as Google Earth or similar software.

### 3.4 Motion Planing

An offline graphical user interface provides a user the ability to select a series of GPS waypoints or generate lawnmower patterns from two-dimensional bounding box. These pre-generated waypoints can be fed into the the gps_goal package so that the vehicle can provide sensor coverage of the desired area. This process is sketched in the GUI to the robot shown in Figure 6D. The GUI provides the user a means to visualize all the necessary information in one window. The top window in the GUI shows the vehicle’s current location and also the waypoints. The user is able to input waypoints in this window. The camera feed and all battery status is also displayed at the bottom left and center respectively. On the right bottom corner, the current orientation of the boat is displayed using a 3D cad model of the vehicle.

As Eddy’s primary task is to monitor infested regions of the lake what is critical to the task is geo-tagged imagery and bathymetric data from the lake. Data is initially stored in a ROS bag and then the data is exported and packaged a KML (Wernecke. (2008)) file so that it may be viewed in context using applications like Google Earth. This data visualization method allows project managers and marine biologists to assess the effectiveness of different removal strategies and the current state of the infestation.

## 4 Ongoing Work

Monitoring and remediating the infestation of EWM at Farlain Lake is an ongoing project. Each spring volunteers head out on the lake to replace markers that were removed in order to save them from ice damage and divers and shore volunteers go into the water to estimate the level of the infestation at the start of the year. Through the summer growing season, plants that have survived the winter grow and new colonies are discovered. An autonomous system that can be used to quantify the nature and magnitude of the infestation will be a significant asset to this ongoing work.

Although operations have been reduced as a consequence of the COVID-19 pandemic, work with Eddy to monitor EWM infestion continues to take place. Work is suspended during the winter months but monitoring i is planned beyond 2020. Bathymetric data coupled with geo-tagged imagery of the infestation will be used directly to estimate the state of the infestation and help inform decisions as to which mechanisms (if any) should be applied to the different colonies.

Each of the various sites identified on Farlain Lake (Figure 1A) is treated differently. Some regions are at a depth in which benthic mats are appropriate. Other regions are too shallow and the bottom too muddy to support this type of treatment. Over the summer of 2020 we plan to traverse each of the areas identified in Figure 1A to provide sensor coverage of the entire area. During this sensor sweep a video of the view beneath the water will be recorded along with the depth of the water column and current robot pose (consisting of GPS location and vehicle orientation). A user interface is being constructed to enable a subject matter expert to quantify the level of infestation given these two signals. Future work will explore using this data to train a deep CNN to perform this assessment automatically, and in future seasons we plan to validate this classifier. The long term goal being to be able to automatically map and assess individual infestations so as to assist in the evaluation of the level of a given infestation and thus help guide treatment options.

Currently information from the sonar and camera is only used as visual information displayed to the user so that they can better evaluate infestation levels. Future work will also explore using the sonar data to estimate the bio-mass of underwater vegetation as well as using the camera to distinguish between EWM and native plant species.

## Data Availability Statement

The raw data supporting the conclusion of this article will be made available by the authors, without undue reservation.

## Ethics Statement

Written informed consent was obtained from the individual(s) for the publication of any potentially identifiable images or data included in this article.

## Author Contributions

RC-D—lead software engineer MJ—principal investigator BBD—lead mechanical engineer JZ—lead technical support EB—software engineer—simulation PA. Technical Leader for the Farlain Lake Community Association’s Integrated EWM Management Plan.

## Funding

This work was supported by the Natural Sciences and Engineering Research Council (NSERC) through the NSERC Canadian Robotics Network (NCRN). This work was also supported by the Canada First Research Excellent Fund (CFREF) through the Vision: Science to Application (VISTA) Doctoral Scholarship program.

## Conflict of Interest

The authors declare that the research was conducted in the absence of any commercial or financial relationships that could be construed as a potential conflict of interest.
